# Cell type composition in bulk prostate cancer tissue is a prognostic biomarker

**DOI:** 10.1016/j.neo.2026.101272

**Published:** 2026-01-13

**Authors:** Xiaokang Zhang, Bjarne Johannessen, Susanne G. Kidd, Mari Bogaard, Ane Stranger, Ulrika Axcrona, Karol Axcrona, Rolf I. Skotheim

**Affiliations:** a Department of Molecular Oncology, Institute for Cancer Research, Oslo University Hospital-Radiumhospitalet, Oslo, Norway; b Department of Pathology, Oslo University Hospital-Radiumhospitalet, Oslo, Norway; c Department of Urology, Akershus University Hospital, Lørenskog, Norway; d Department of Informatics, Faculty of Mathematics and Natural Sciences, University of Oslo, Oslo, Norway

**Keywords:** Cell type composition, Machine learning, Molecular subtyping, Multifocality, Prognostic biomarker, Prostate cancer

## Abstract

Prostate cancer, among the most prevalent cancer types globally, exhibits marked heterogeneity and varying disease progression and clinical outcomes. Improved molecular subtyping is needed for patient stratification. Since prostate cancer has relatively few somatic point mutations, whole-transcriptome data instead offers a rich and relevant source of molecular data. We analyzed bulk tissue transcriptomes from four cohorts to characterize primary prostate cancer’s cell type composition. A deconvolution of cell types was performed based on gene expression profiles. Patients with available multi-sample regional data from different tumor foci were analyzed for intrapatient heterogeneity. Three cell type composition subtypes were defined: T cells enriched (TCE), epithelial cells enriched (EPCE), and tumor-associated stromal cells enriched (TASCE). A machine learning model was developed to classify these subtypes and validated in three independent cohorts. The subtyping demonstrated a high correlation with established clinicopathological parameters (e.g., Gleason score, p-value < 0.05), and the classifier showed a promising ability to predict biochemical recurrence. Moreover, our analysis revealed that interfocal heterogeneity in patients with multifocal cancer significantly surpassed intrafocal heterogeneity (p-value < 0.05). In conclusion, this study provides a novel prostate cancer subgrouping based on cell type composition, with the TASCE subtype significantly associated with high biochemical recurrence risk.

## Highlight

• Cellular composition subtypes are defined for prostate cancer.
• Robust classifier trained and validated to predict subtypes across independent cohorts.
• Interfocal heterogeneity exceeds intrafocal heterogeneity in subtyping.
• Subtyping highly correlates with biochemical recurrence.

## Introduction

Prostate cancer is one of the most common cancer types affecting men worldwide, posing a significant clinical challenge and public health concern [1]. Despite advances in diagnostic methods and treatment strategies, stratification of localized prostate cancer to treatment strategies and accurately predicting patient outcomes remain grand challenges. An important research area in prostate cancer is thus enhancing the prognostication of patients with localized disease [2]. This would aid clinical decisions on who should undergo active surveillance and who should receive radical treatment – and for the latter, who would benefit from intensified treatment.

Molecular characterization of prostate cancer is complicated by the fact that most patients have multiple primary tumor foci upon diagnosis [3]. These tumors are both inter- and intrafocally heterogeneous, meaning that there is a coexistence of cells with different phenotypic and genomic characteristics across and within tumor foci [[4], [5], [6], [7], [8], [9], [10]]. Patients treated with radical prostatectomy experience biochemical relapse (BCR) of the disease at approximately 20%−40% [11]. RNA-level data, including expression of protein-coding genes, long noncoding RNAs, and gene fusions, have been extensively studied for the development of molecular prognostic biomarkers for prostate cancer [5,[12], [13], [14], [15], [16]]. The emergence of spatial transcriptomics has revealed a new landscape of heterogeneity in prostate cancer [[17], [18], [19]]. Yet, most transcriptome-wide data exist on bulk tissue [4,[20], [21], [22]].

Despite these efforts, the contribution of the tumor microenvironment (TME) to prostate cancer’s clinical behavior and its potential as a prognostic tool remains relatively underexplored. The TME encompasses an array of non-malignant cells and extracellular components that interact with tumor cells in further development of the cancer ultimately leading to a metastatic state [23]. Further, the TME profoundly impacts therapeutic responses, particularly with the advent of immunotherapy [24]. While immunotherapy has shown remarkable efficacy in other cancer types, the proportion of prostate cancer patients benefiting remains modest [[25], [26], [27], [28]]. The inherent heterogeneity of the TME could possibly explain this limited response [29,30].

Prostate cancer cells express immunosuppressive cytokines and growth factors, contributing to an immunosuppressive TME that is generally filled with protumorigenic tumor-associated macrophages, regulatory T cells, and myeloid-derived suppressor cells [31]. However, the existence of functional and anti-tumor reactive tumor-infiltrating lymphocytes (TILs) has been demonstrated through their isolation and expansion from primary prostate cancer tissues [32].

Prostate cancer is considered an immunologically “cold” tumor due to its sparse T-cell infiltration and weak response to checkpoint inhibitor monotherapies [33,34]. Studies on spatial and molecular immune profiling have attempted to define the immune landscape of various cancers [30], classifying prostate cancer within the “inflammatory” subtype, characterized by elevated expression of Th17- and Th1-associated genes. Nevertheless, a comprehensive classification of the prostate cancer TME is still needed [35].

In this study, we explored the cell type composition of prostate cancer utilizing whole-transcriptome RNA sequencing (RNA-seq) data from bulk tumor samples sourced from The Cancer Genome Atlas (TCGA) Research Network [20]. By estimating the fractions of various cell populations, including immune cells, stromal cells, and cancer cells themselves, we stratified patients into distinct immune-related subtypes and assessed associations with time to BCR. Moreover, we trained a machine learning model to classify these subtypes and tested its predictive accuracy using RNA-seq data from three independent prostate cancer cohorts.

## Materials and methods

### RNA sequencing datasets

We analyzed four cohorts with bulk tissue RNA-seq data from prostate cancer patients treated with radical prostatectomy, where RNA was isolated from fresh-frozen or formalin-fixed and paraffin-embedded (FFPE) tissue samples. From all four cohorts, the data were genome-scale, and with expression estimates on the gene level. From three of the cohorts, expression data were available from one sample per patient, and from one cohort, data from multiple samples per patient were analyzed. The four datasets are referred to as the TCGA, Hamburg, Atlanta, and Oslo datasets, respectively (Table 1).

**Table 1:** Datasets with genome-scale and gene-level expression values from RNA-sequencing of prostate cancer tissue samples.

**Dataset name**	**Patients**	**Tissue samples**	**Tissue**	**Age (mean)**	**Follow-up time (**m**onths,** m**edian)**	**BCR time (months, mean)**
TCGA	405	405	Fresh frozen	61	20.5	16.6
Hamburg	82	82	Fresh frozen	47	36.1	33.1
Atlanta	106	106	FFPE	61	68.5	23.4
Oslo	23 (64)*	64	Fresh frozen	62	115.8	40.1

⁎ Multisample dataset, two-to-three malignant tissue samples included from each of 23 prostate cancer patients, totaling 64 malignant tissue samples. Abbreviations: TCGA, The Cancer Genome Atlas; FFPE, formalin-fixed and paraffin-embedded.

The TCGA dataset, downloaded using the R packages “TCGAbiolinks” [[36], [37], [38]] and “RTCGAToolbox” [39], includes gene expression profiles (in transcripts per million, TPM) from one fresh-frozen tissue sample from each of the 550 prostate cancer patients [20] and clinical information from 500 patients. There are 405 patients with both gene expression profiles and clinical information, and 52 of them experienced BCR.

The Hamburg dataset [21], downloaded from cBioPortal (DKFZ2018) [40], includes the gene expression profiles (in reads per kilobase of transcript per million mapped reads, RPKM) from one prostate cancer tissue sample from each of the 95 patients (fresh-frozen tissues; for the six patients with multiple samples, the first sample based on the index was kept as representative of that patient), and clinical information from 269 patients. There are 82 patients with both gene expression profiles and clinical information, and 18 of them experienced BCR.

The Atlanta dataset [22], downloaded from Gene Expression Omnibus (GSE54460), includes gene expression profiles (in fragments per kilobase of transcript per million mapped reads, FPKM) from one prostate cancer tissue sample from each of the 106 patients (FFPE tissues) and their clinical information. Among the 106 patients, 55 have experienced BCR.

The Oslo dataset [4] was generated in-house and includes gene expression profiles (in transcripts per million, TPM) and whole-exome sequencing [7] from 64 fresh-frozen tissue samples from 23 patients and their clinical information [4,7]. The sample set includes two to three malignant tissue samples from at least two spatially separated malignant foci, in addition to one patient with two samples from a single malignant focus. Among the 23 patients, 5 have experienced BCR. These samples and patients were selected from a total cohort of 571 prostate cancer patients treated with radical prostatectomy between 2010 and 2012 at Oslo University Hospital-*Radiumhospitalet* [4]. The profiled sub cohort only included patients with multifocal prostate cancer, as described previously [7]. Using whole-exome sequencing data from patients in the Oslo cohort from our previous publication [7], we estimated tumor purity with the R package “PurBayes” with default settings [41].

### Cell type composition via deconvolution of bulk tissue gene expression

From the four RNA-seq datasets, proportions of seven different cell types were estimated by the EPIC (Estimation of Proportions of Immune and Cancer cells) algorithm [42]. These populations encompass B cells, CD4+ *T* cells, CD8+ *T* cells, natural killer (NK) cells, macrophages, endothelial cells, and cancer-associated fibroblasts (CAFs). Additional cells not accounted for by these seven categories were aggregated under the designation “uncharacterized cells” in the TME. This designation is expected to predominantly comprise cancer cells and non-malignant epithelial cells.

The cell type composition was represented by the proportion of each cell type relative to the total cellular constituency within the tumor sample. The Oslo dataset, which included multiple samples per patient and per focus, was analyzed to assess three types of heterogeneity: interpatient (pairs of samples obtained from different patients), interfocal (pairs of samples obtained from different tumor foci within the same patient), and intrafocal (pairs of samples obtained from the same tumor focus) heterogeneity. We first performed a Z-score transformation on the estimated cell type proportions for each sample and then used Cosine similarity to measure the similarity of each pair. The statistical differences between the three types were assessed using the Wilcoxon rank-sum test.

### Clustering of subgroups and construction of classifier

To identify potential patient subgroups, we applied unsupervised clustering to the proportions of the different cell types. Prior to clustering, we performed Z-transformation of the original cell type proportions to normalize the dataset. Subsequently, resampling-based consensus clustering [43,44] incorporating partitioning around medoids was utilized.

To validate the stromal phenotype of the identified subtypes, we examined the expression levels of nine established Cancer-Associated Fibroblast (CAF) markers (including *ACTA2, FAP, PDGFRB, POSTN, COL1A1, FN1, VIM, DCN*, and *LUM*), comparing their abundance across the subtypes. Following this validation, we performed a comprehensive differential expression analysis using the R package “limma” [45] to elucidate the biological distinctions between the subtypes. Gene expression data was log2-transformed and filtered to remove low-expression genes, and linear models were employed to identify differentially expressed genes through pairwise comparisons. To uncover functional implications, Gene Set Enrichment Analysis (GSEA) was subsequently performed using the R package “clusterProfiler” [46]. Genes were ranked by their t-statistics derived from the differential expression analysis, and enrichment was evaluated against Gene Ontology (GO) terms and Kyoto Encyclopedia of Genes and Genomes (KEGG) pathways, with significance defined by a Benjamini-Hochberg adjusted p-value < 0.05.

To construct a classifier for the subgroups obtained from consensus clustering, we trained a linear discriminant analysis (LDA) classification model using the cell type composition as input. With only eight features (cell types), a simple linear model rather than a complex nonlinear model is preferred. Previous studies demonstrate the efficacy of LDA in predicting prognosis in cancer research [[47], [48], [49], [50]]. The classifier was trained on the TCGA dataset, using the function “lda” from the R package “MASS” (version 7.3-54). We validated the predictive capability of this trained model on the three other independent datasets.

## Results

### Identification, training, and validation of prostate cancer cell type composition subtypes

Using RNA-seq data from 405 prostate cancer samples in the TCGA cohort, we employed the EPIC algorithm to determine the proportions of eight different cell types within each sample (detailed cell type compositions are provided in Supplementary Table 1).

**Fig. 1:**
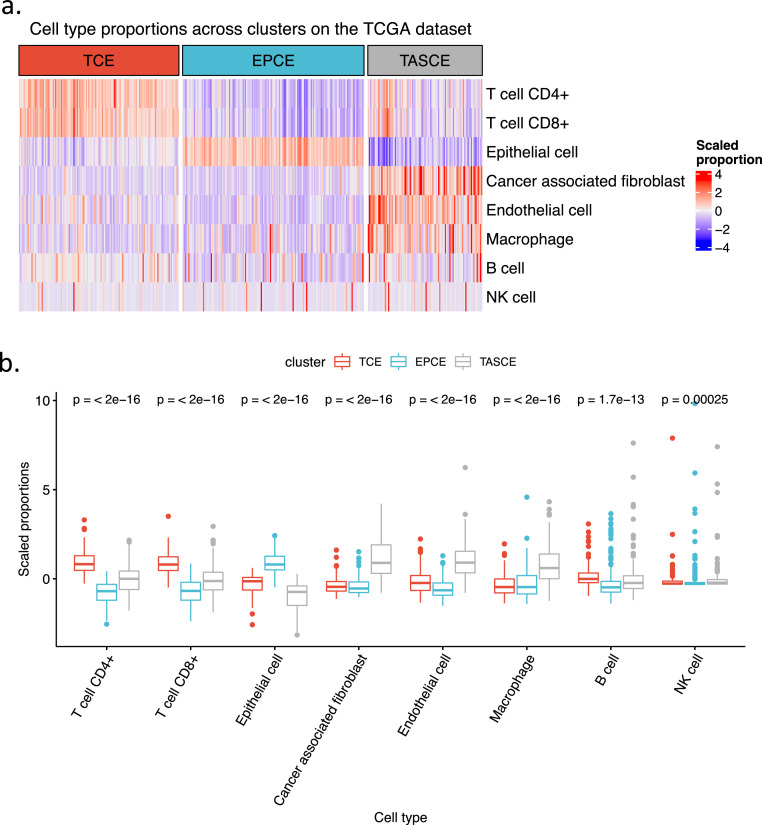
**Prostate cancer cell type composition subtypes.** (a) Heatmap of estimated cell types compositions across the three clustered groups. (b) Boxplot of each cell type’s scaled proportions among the three groups. The scaled proportions are Z-transformed percentages of each cell type. Abbreviations: EPCE, epithelial cells enriched; TASCE, tumor-associated stromal cells enriched; TCE, T cells enriched; TCGA, The Cancer Genome Atlas.

The clustering analysis arranged the patients into three subtypes based on their predominant cell type compositions (Supplementary Fig. 1). We named these T cells enriched (TCE), epithelial cells enriched (EPCE), and tumor-associated stromal cells enriched (TASCE). These groupings were visually represented via a heatmap (Fig. 1a). Notably, each of the eight cell types exhibited significantly disparate distributions across the three identified cell type composition subtypes, with all comparisons yielding p-values <0.0003 (Fig. 1b).

To validate the biological interpretation of the TASCE subtype as stroma-rich and biologically active, we analyzed the expression of specific stromal markers and performed functional pathway enrichment analyses. Examination of nine canonical CAF markers revealed that their expression was consistently higher in the TASCE subtype compared to the TCE and EPCE subtypes (Supplementary Fig. 2), providing direct evidence of enhanced stromal presence. This observation was further substantiated by GSEA. In comparisons between TASCE and TCE, we observed a marked enrichment of pathways and biological processes associated with extracellular matrix (ECM) organization and remodeling. GO analysis identified key terms such as "collagen fibril organization", "collagen metabolic process", and "extracellular matrix structural constituent" (Supplementary Fig. 3), while KEGG analysis highlighted the upregulation of "Focal adhesion", "ECM-receptor interaction", and "Cell adhesion molecules" (Supplementary Fig. 4). Additionally, the TASCE subtype exhibited enrichment in immune and inflammatory signaling pathways, including "NF-kappa B signaling", "Cytokine-cytokine receptor interaction", and "adaptive immune response". These functional signatures, which were consistently observed in comparisons against other subtypes, demonstrate that TASCE tumors are characterized by active stromal remodeling and complex microenvironmental signaling.

Additionally, the EPIC algorithm was employed to estimate the proportions of the same eight cell types across the other three datasets (Supplementary Tables 2-4). The correlation between tumor purity and the proportion of epithelial cells was investigated on the Oslo dataset, revealing a weak yet significant linear positive correlation (Pearson correlation coefficient = 0.34, p-value = 0.007; Supplementary Fig. 5).

We trained an LDA classifier using cell type composition data from the TCGA dataset. The performance of this classifier was then validated using the other three independent datasets. Visualizations of the classification results, presented as heatmaps (Supplementary Fig. 6), demonstrated consistent patterns of cell type predominance within each subtype across these validation datasets, except for macrophages in the TASCE subtype within the Hamburg dataset, where they were less enriched than in the other datasets.

### Heterogeneity of the cell type composition subtypes

**Fig. 2:**
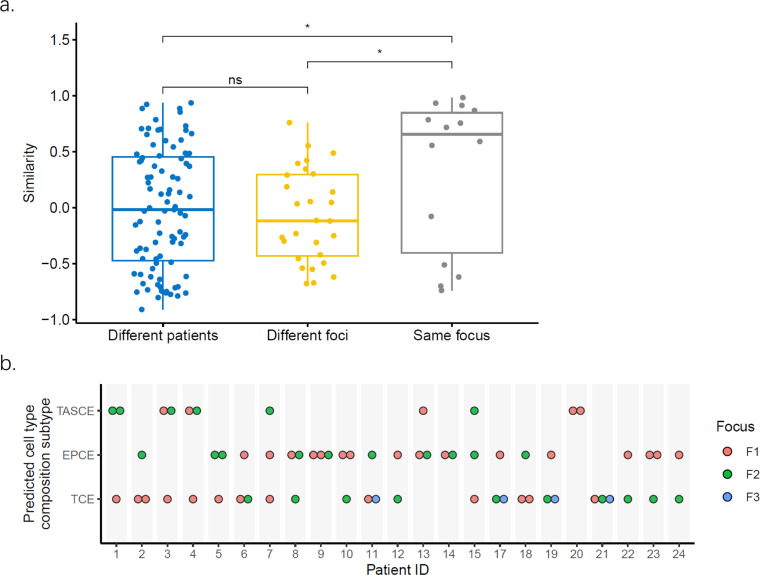
**Heterogeneity of the prostate cancer cell type composition.** (a) Interpatient, interfocal, and intrafocal heterogeneity of the cell type composition. The similarity (y-axis) is represented by cosine similarity. ns: *p* > 0.05, * *p* ≤ 0.05 (Wilcoxon rank-sum test). (b) Inter and intrafocal heterogeneity of the cell type composition subtyping.

We conducted a comprehensive analysis to evaluate the heterogeneity among the identified cell type composition subtypes, focusing on three specific types of heterogeneity: interpatient, interfocal, and intrafocal heterogeneity (Fig. 2a). Our investigations revealed that the cell type composition similarity was substantially higher within a single tumor focus compared to similarity across different foci or patients, but no significant difference was observed between the latter two, which implied notable interfocal heterogeneity, whereas intrafocal heterogeneity was considerably lower.

The predicted cell type composition subtype for each sample in the Oslo multifocality dataset is shown in Fig. 2b. It was relatively uncommon for all samples from the same patient to be consistently categorized into a single subtype. Specifically, among the 23 patients, only four patients (patients 9, 14, 20, and 21) had all samples classified into a single subtype. Conversely, among the 15 patients who had data from multiple samples from the same focus, up to 8 cases had all samples from an individual focus falling uniformly into the same subtype. These findings suggest that cell type composition subtyping exhibits less heterogeneity at the intrafocal level than that at the interfocal level.

### Cell type composition subtypes and clinicopathological parameters

**Fig. 3:**
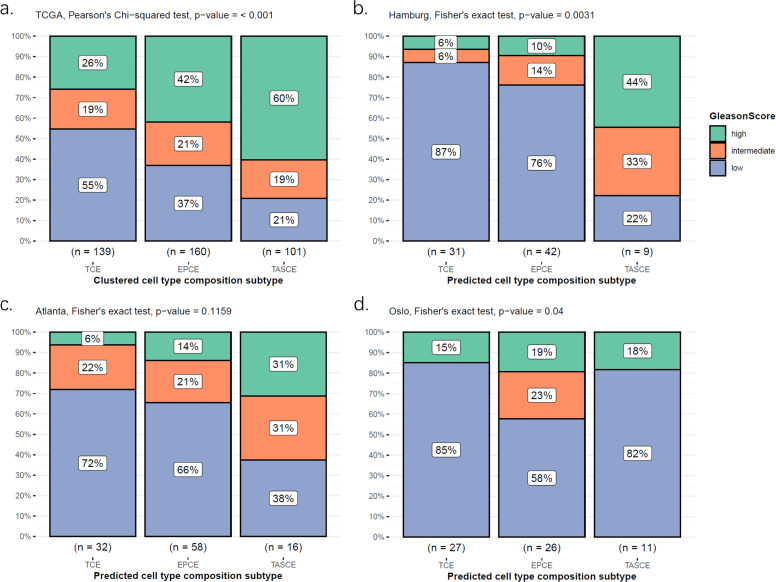
**Association between cell type composition subtypes and Gleason score.** (a) The TCGA dataset, (b) the Hamburg dataset, (c) the Atlanta dataset, and (d) the Oslo dataset. Gleason scores are at the patient level in panels (a-c), and at the sample level in panel (d) for the Oslo dataset.

The association between the subtypes and clinicopathological parameters (*e.g.* Gleason score and pathological tumor stage [pT stage]) was studied across the four cohorts. The Gleason scores were divided into three categories for this analysis: 4 + 4 and 4 + 5 or above were assigned to “high-grade”, 4 + 3 was assigned to “intermediate-grade”, and the remaining scores were assigned to “low-grade”. Fig. 3 illustrates the distributions of patients or samples with different Gleason scores in the three cell type composition subtypes within each cohort. It was consistently observed that high-grade tumors were predominantly found in the TASCE subtype, whereas the TCE subtype was more commonly associated with low-grade tumors. It is noteworthy that the Oslo dataset provided the Gleason score on a per sample basis, as depicted in Fig. 3d. This, along with the selection criteria for multifocality, results in a high fraction of low-grade samples.

We also examined the relationship between cell type composition subtypes and pT stage. The results indicated that higher pT stages were more frequently associated with the TASCE subtype, whereas lower pT stages were associated with the TCE subtype (Supplementary Fig. 7). For this analysis, the Oslo dataset was excluded as the cell type composition subtypes are predicted on the sample level, whereas the pT stage was scored on the patient level.

Overall, there is a clear trend across the three cell type composition subtypes: patients or samples characterized by the TCE subtype exhibit both lower Gleason scores and pT stages, indicating a potential correlation with less aggressive and less advanced disease. Conversely, patients or samples with the TASCE subtype often present with higher Gleason scores and pT stages, suggestive of a more aggressive and advanced disease.

### The cell type composition subtype is a predictor of biochemical recurrence

**Fig. 4:**
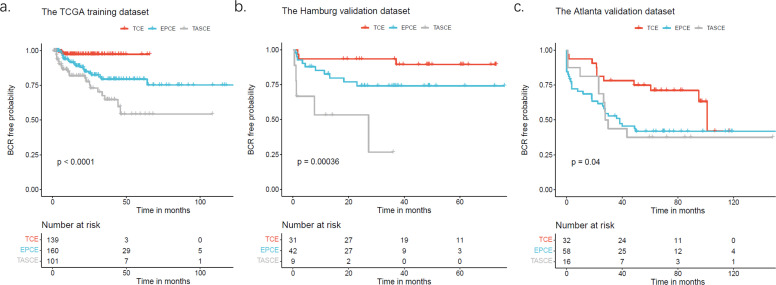
**Cell type composition subtyping is significantly associated with biochemical relapse.** (a) The TCGA dataset as a training cohort, (b) Hamburg and (c) Atlanta as validation cohorts. Kaplan–Meier plots were truncated when the number at risk was less than five. Abbreviations: BCR, biochemical recurrence.

Given the observation that higher Gleason scores and pT stages correlate with advanced and aggressive cancers, we hypothesized that the TCE subtype would be associated with a more favorable prognosis after radical prostatectomy, whereas the TASCE subtype would indicate a poorer outcome. Utilizing BCR as an endpoint, time-to-event analysis highlighted distinct prognostic outcomes among the three subtypes (p-value < 0.0001; Fig. 4a). In the validation phase, the analysis was confined to the Atlanta and Hamburg datasets due to the limited number of BCR events in the Oslo dataset. Similarly to the results from the TCGA dataset, significant associations between subtypes and BCR were identified in the validation cohorts, with the TCE subtype having the most favorable prognosis and the TASCE subtype having the worst prognosis (p-value = 0.0004 and p-value = 0.04; Fig. 4, Fig. 4).

We applied univariable Cox regression models to individually evaluate the association of cell type composition subtype, Gleason score, and pT stage with BCR, respectively, and found that all three parameters were significantly associated with BCR (Table 2). Subsequent multivariable Cox regression analysis incorporating all three parameters revealed that cell type composition subtypes had superior predictive capability over Gleason score and pT stage (Table 2).

**Table 2:** Univariable and multivariable Cox regression models on TCGA cohort with biochemical relapse as endpoint. All the Cox regression analyses have met the assumption of proportional hazards.

	**Univariable**		**Multivariable**	
	HR (95% CI)	P-value	HR (95% CI)	P-value
**Cell type composition subtypes**				
TCE	Reference			
EPCE	5.3 (1.6-17.6)	0.007*	3.9 (1.2-13.2)	0.027*
TASCE	10.9 (3.3-36.2)	<0.001*	6.0 (1.8-20.6)	0.004*
**Gleason** s**core**				
Low (≤ 7 = 3 + 4)	Reference			
Intermediate (7 = 4 + 3)	5.2 (1.6-16.8)	0.006*	3.2 (1.0-11.0)	0.059
High (≥ 8)	8.9 (3.2-25.0)	<0.001*	3.4 (1.1-11.7)	0.034*
**pT stage**				
T2	Reference			
T3a	5.1 (1.7-15.1)	0.003*	2.9 (1.0-9.0)	0.061
T3b	9.6 (3.4-27.6)	<0.001*	3.9 (1.2-12.2)	0.021*

⁎ Statistically significant, *p* < 0.05. Abbreviations: TCE, T cells enriched; EPCE, epithelial cells enriched; TASCE, tumor-associated stromal cells enriched; HR, hazard ratio; CI, confidence interval; pT stage, pathological tumor stage. The assumption of proportional hazards was met for all fitted models.

To further explore the prognostic utility, we constructed multivariable Cox regression models integrating various parameters and assessed the changes in concordance index. By adding pT stage and cell type composition subtype to the regression model initially fitted with Gleason score, we observed distinct improvements in predictive power. Specifically, the concordance index was 0.677 for the model with Gleason score alone. Incorporating pT stage into the model raised the index to 0.722, while the integration of cell type composition subtype increased it to 0.742. When both pT stage and cell type composition subtype were added, the model achieved a concordance index of 0.76 (Supplementary Fig. 8). These enhancements, particularly with the addition of cell type composition subtype, underscore its significance as a valuable predictive marker for BCR.

## Discussion

In this study, we explored the prognostic potential of cell type composition subtypes from radical prostatectomy samples, and their relationship with established clinicopathological features and patient outcomes. The results from our analysis underscore the significance of cellular heterogeneity in the TME, and the utility of cell type composition subtypes as prognostic biomarkers for BCR, a crucial clinical endpoint in prostate cancer management. The concept of reactive stroma (also named as wound repair stroma) as an important factor for prostate cancer progression was recognized >20 years ago [51] and has further been demonstrated as an important factor for the prognosis for prostate cancer patients [52].

Previous work by Sturm et al. benchmarked seven state-of-the-art computational methods estimating the composition of immune, stromal, tumor and other cell types that comprise the microenvironment of malignant tissues [53]. The evaluated methods [42,[54], [55], [56], [57], [58], [59]] demonstrated high accuracy for well-defined cell-type signatures on simulated bulk RNA-seq samples derived from a single-cell RNA-seq dataset and on true bulk RNA-seq samples. These methods can be conceptually categorized into two groups: marker-gene-based and deconvolution-based approaches. The output scores are either arbitrary units or cell fractions, allowing for intra- or inter-sample comparisons, or both. Only two of these methods, EPIC [42] and quanTIseq [54] output absolute cell fractions, facilitating a more intuitive and practical interpretation by enabling comparisons both within and between samples. The high stability of EPIC, particularly evident within the TCGA dataset, led to its selection in our investigation.

Using the gene expression profiles derived from bulk RNA-seq data, we estimated the cell type composition of prostate cancer via deconvolution algorithms. This enabled the stratification of patients within the TCGA cohort into three distinct subgroups, each defined by its enriched cell type. Using machine learning techniques, a predictive classifier was generated and subsequently validated across three independent datasets. The three subtypes exhibited heterogeneity both interpatiently and interfocally, but less so intrafocally. The cell type composition subtypes showed a strong correlation with significant pathological parameters and are robust predictors of BCR – an association consistently validated across two independent datasets.

Our multivariable analysis particularly demonstrated the superior prognostic capacity of cell type composition subtypes relative to traditional parameters like Gleason score and pT stage. The predictive accuracy added by these subtypes underscores the intricate interplay between the TME and tumor behavior, presenting a compelling case for integrating cellular composition analysis into the clinical decision-making framework for prostate cancer patients.

The current study's stratification into three cell type composition subtypes reflects underlying biological processes that may influence prostate cancer progression and response to therapy. The TCE subtype, enriched with both CD8+ and CD4+ *T* cells, suggests that a robust anti-tumor immune response can translate to a favorable prognosis in prostate cancer. CD8+ *T* cells are critical cytotoxic components attacking and eliminating cancer cells, whereas CD4+ *T* cells sustain these cytotoxic responses and prevent exhaustion of CD8+ *T* cells [60]. Whereas contrasting prognoses have been reported concerning CD8+ *T* cell infiltration in prostate cancer [61], the synergistic effect of CD8+ and CD4+ *T* cells remains less explored [62]. Our findings appear to contradict Grogg et al.'s study which did not find a correlation between preoperative inflammatory markers and adverse outcomes [63]. This suggests that nuanced differences in immune cell populations, rather than broad inflammation, are critical for prognostication. Patients with TCE subtype cancers showed a tendency toward more favorable outcomes, aligning with our understanding of the anti-tumor immune environment.

The EPCE subtype, enriched with uncharacterized cells, mainly consists of epithelial cells in prostate cancer. While there was an initial concern that the classification into this subtype might inadvertently reflect tumor purity, our analysis showed that this correlation, while present, was moderate.

The TASCE subtype, enriched with stroma-associated cell types, indicates more complex interaction with the tumor environment. Tumor-associated stromal cells derive from various sources, including fibroblasts and endothelial cells undergoing endothelial-mesenchymal transition (EndMT), and macrophages [64]. Cancer-associated fibroblasts (CAFs) are a specialized group of fibroblasts involved in tumor growth and invasion [65]. Besides normal fibroblasts, endothelial cells have also recently been recognized as an important origin of CAFs [64]. EndMT disrupts endothelial junctions and the blood barrier, facilitating cancer cell intravasation – a process also promoted by tumor associated macrophages [66]. This intricate cellular crosstalk, detailed by Ronca et al. [67], emphasizes stromal cells’ proactive role in tumor progression. Furthermore, high levels of reactive stroma have been linked to an increased risk of cancer-specific mortality in prostate cancer [68]. Thus, patients categorized under the TASCE subtype might be at increased risk for developing aggressive disease and worse prognosis. This subtype's association with negative clinical outcomes is consistent with its cellular composition's propensity to support tumor growth and spread.

In the cellular intricacies of the TME, myeloid lineage cells have recently surfaced as important players in prostate cancer progression. Recent findings by Guo et al. [69] highlight that myeloid inflammation is associated with resistance to androgen receptor signaling inhibitors, adding another dimension to our understanding of prostate cancer biology. Recent spatial transcriptomics investigations have also revealed a distinct club-like cell subtype in prostate cancer, characterized by a senescence-associated secretory phenotype and increased myeloid-derived suppressor cell activity, which may contribute to treatment resistance [19]. A limitation in our analytical approach lies in the categorization of myeloid lineage cells; while our classification effectively captures macrophages linked to prostate cancer progression, other myeloid lineage cells are not specifically identified. Another assumption made is that uncharacterized cells consist primarily of cancer cells and benign epithelial cells. This assumption may be overly simplistic, as it could encompass cells of non-epithelial origin as well.

While our study identified distinct cellular composition subtypes and their value in prognostic stratification of prostate cancer patients, it underscores the need for further comprehensive characterization of the TME, specifically the myeloid component. The development of more refined deconvolution algorithms, alongside single-cell analyses, spatial transcriptomics, flow cytometry, and immunohistochemistry, will enable a nuanced understanding of the tumor milieu and its relationship with disease progression and therapy response, paving the way for more precise, personalized therapeutic strategies in prostate cancer.

In conclusion, this study emphasizes the powerful potential of tumor cellular composition to provide biological insights and prognostic stratification in prostate cancer. The TASCE subtype, enriched for tumor-associated stromal cells, showed a clear association with aggressive tumor phenotypes. It is clinically promising, as multivariable Cox regression revealed, that the TASCE subtype has a superior predictive capability over Gleason score and pT stage. Cell type composition analyses are a promising approach for personalized prognostication and treatment strategies in prostate cancer, potentially leading to more tailored therapeutic interventions and thereby improved patient outcomes.

## Data availability

The TCGA, Hamburg and Atlanta datasets are publicly available and were downloaded as described in Materials and methods. As for the Oslo dataset, it is available from the corresponding author upon reasonable request. The source codes are available at https://github.com/zhxiaokang/ProcTIME.

## Additional information

Supplementary Table 1. The detailed cell type compositions estimated from the TCGA dataset.

Supplementary Table 2. The detailed cell type compositions estimated from the Hamburg dataset.

Supplementary Table 3. The detailed cell type compositions estimated from the Atlanta dataset.

Supplementary Table 4. The detailed cell type compositions estimated from the Oslo dataset.

Supplementary Fig. 1. Consensus clustering results on the TCGA dataset based on the cell type composition. (a) Consensus matrix for three clusters (*k* = 3). (b) The cumulative distribution functions of the consensus matrix for each k (number of clusters). The clean Consensus matrix plot in (a) and the stable distribution with *k* = 3 in (b) indicate that the optimal number of clusters is three. Abbreviation: CDF, cumulative distribution function.

Supplementary Fig. 2. Expression of Cancer-Associated Fibroblast (CAF) markers across three clusters of the TCGA dataset. * *p* ≤ 0.05, **** *p* ≤ 0.0001 (Wilcoxon rank-sum test).

Supplementary Fig. 3. Gene Set Enrichment Analysis (GSEA) of Gene Ontology (GO) terms enriched in TASCE versus TCE subtypes.

Supplementary Fig. 4. Gene Set Enrichment Analysis (GSEA) of KEGG pathways comparing TASCE and TCE subtypes.

Supplementary Fig. 5. The correlation between the tumor purity and the proportion of epithelial cells on the Oslo dataset.

Supplementary Fig. 6. Prediction of cell type composition subtype on the Hamburg dataset (a), the Atlanta dataset (b), and the Oslo dataset (c). To be noted, the columns in (a) and (b) are patients, but are tissue samples in (c).

Supplementary Fig. 7. Association between cell type composition subtype and pathological stage in the TCGA dataset (a), the Hamburg dataset (b) and the Atlanta dataset (c). Abbreviation: pT stage, pathological stage.

Supplementary Fig. 8. Concordance indices of Cox regression models fitted with selected clinicopathological variables and cell type composition subtype. Abbreviation: pT stage, pathological stage.

## CRediT authorship contribution statement

**Xiaokang Zhang:** Writing – review & editing, Writing – original draft, Visualization, Software, Methodology, Investigation, Formal analysis, Data curation. **Bjarne Johannessen:** Writing – review & editing, Software, Methodology, Data curation. **Susanne G. Kidd:** Writing – review & editing, Investigation. **Mari Bogaard:** Writing – review & editing, Investigation. **Ane Stranger:** Writing – review & editing, Investigation. **Ulrika Axcrona:** Writing – review & editing, Investigation. **Karol Axcrona:** Writing – review & editing, Investigation. **Rolf I. Skotheim:** Writing – review & editing, Writing – original draft, Supervision, Resources, Project administration, Investigation, Funding acquisition, Conceptualization.

## Declaration of competing interest

The authors declare that they have no known competing financial interests or personal relationships that could have appeared to influence the work reported in this paper.

## Appendix Supplementary materials

Image, application 1

Image, application 2

Image, application 3

Image, application 4

Image, application 5

Image, application 6

Image, application 7
