# Supplementary material for: Cell type composition in bulk prostate cancer tissue is a prognostic biomarker
Source: Neoplasia. 2026 Jan 13;72:101272. doi: 10.1016/j.neo.2026.101272 (PMC12828772; doi:10.1016/j.neo.2026.101272)
Supplement: Supplementary file 6 [file mmc6.pdf]

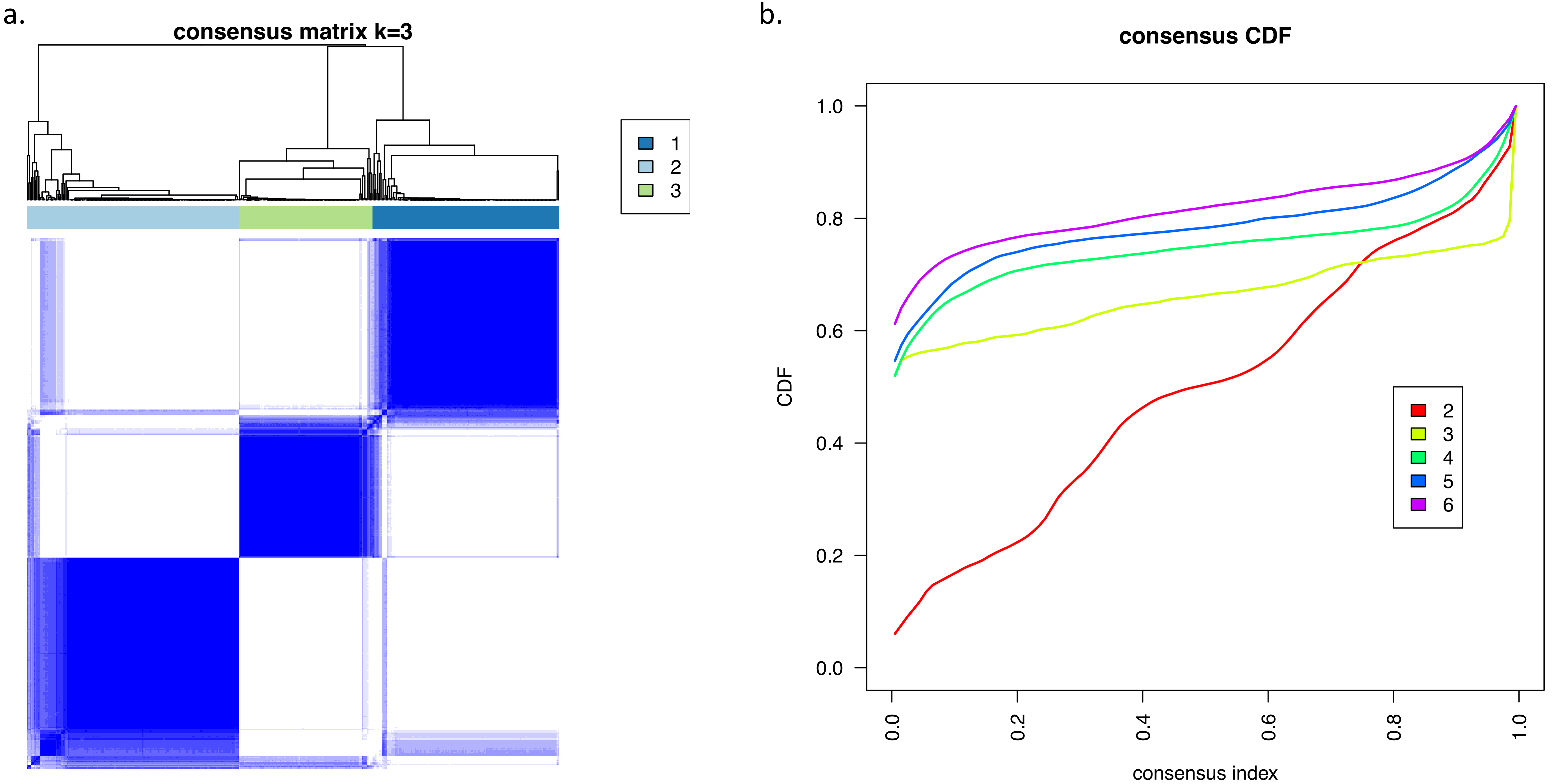
a.
consensus matrix k=3
1
2
3
b.
consensus CDF
CDF
consensus index
0.0
0.2
0.4
0.6
0.8
1.0
0.0
0.2
0.4
0.6
0.8
1.0
2
3
4
5
6

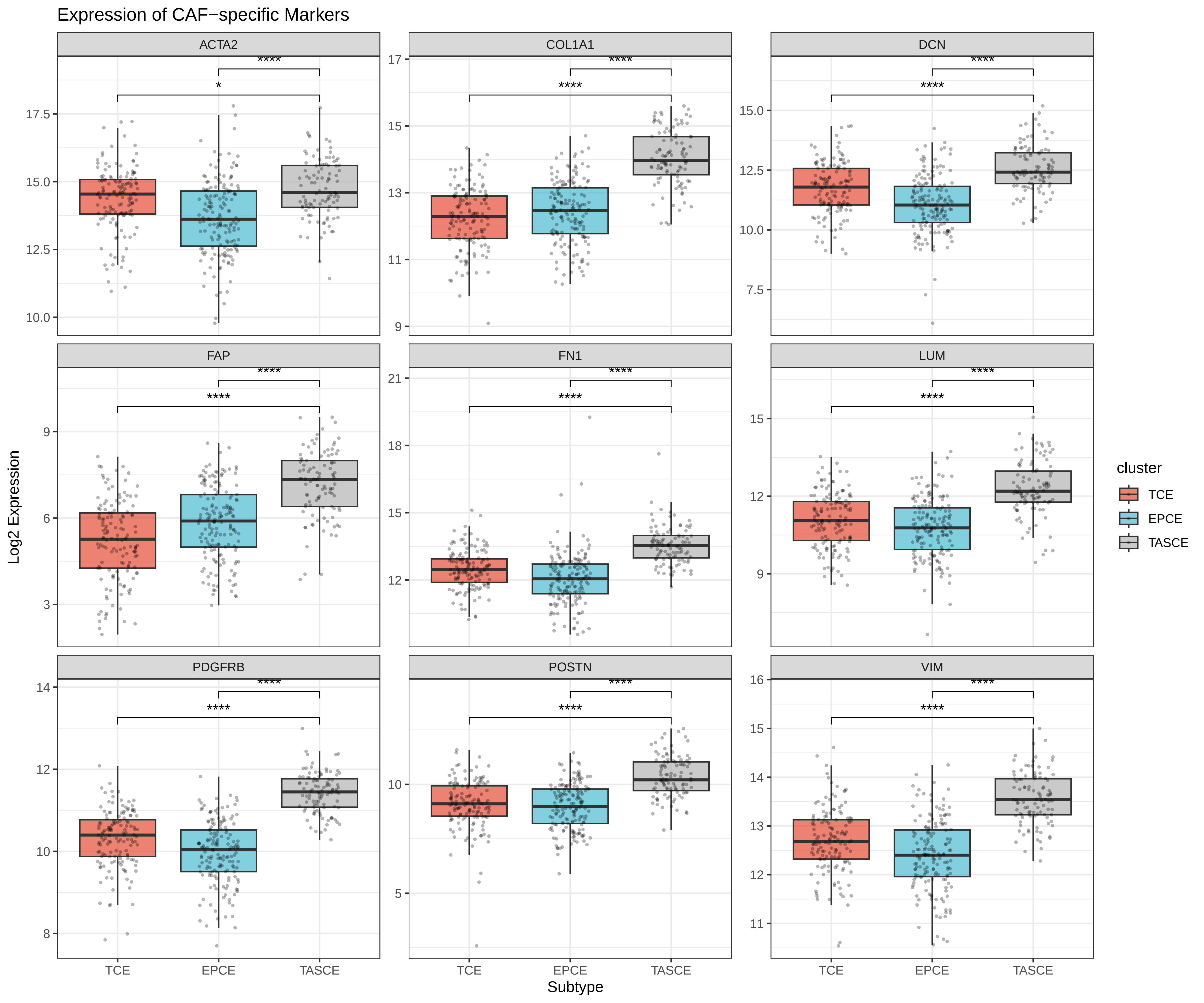

Expression of CAF−specific Markers
ACTA2
COL1A1
DCN
FAP
FN1
LUM
PDGFRB
POSTN
VIM
Log2 Expression
Subtype
TCE
EPCE
TASCE
cluster
TCE
EPCE
TASCE

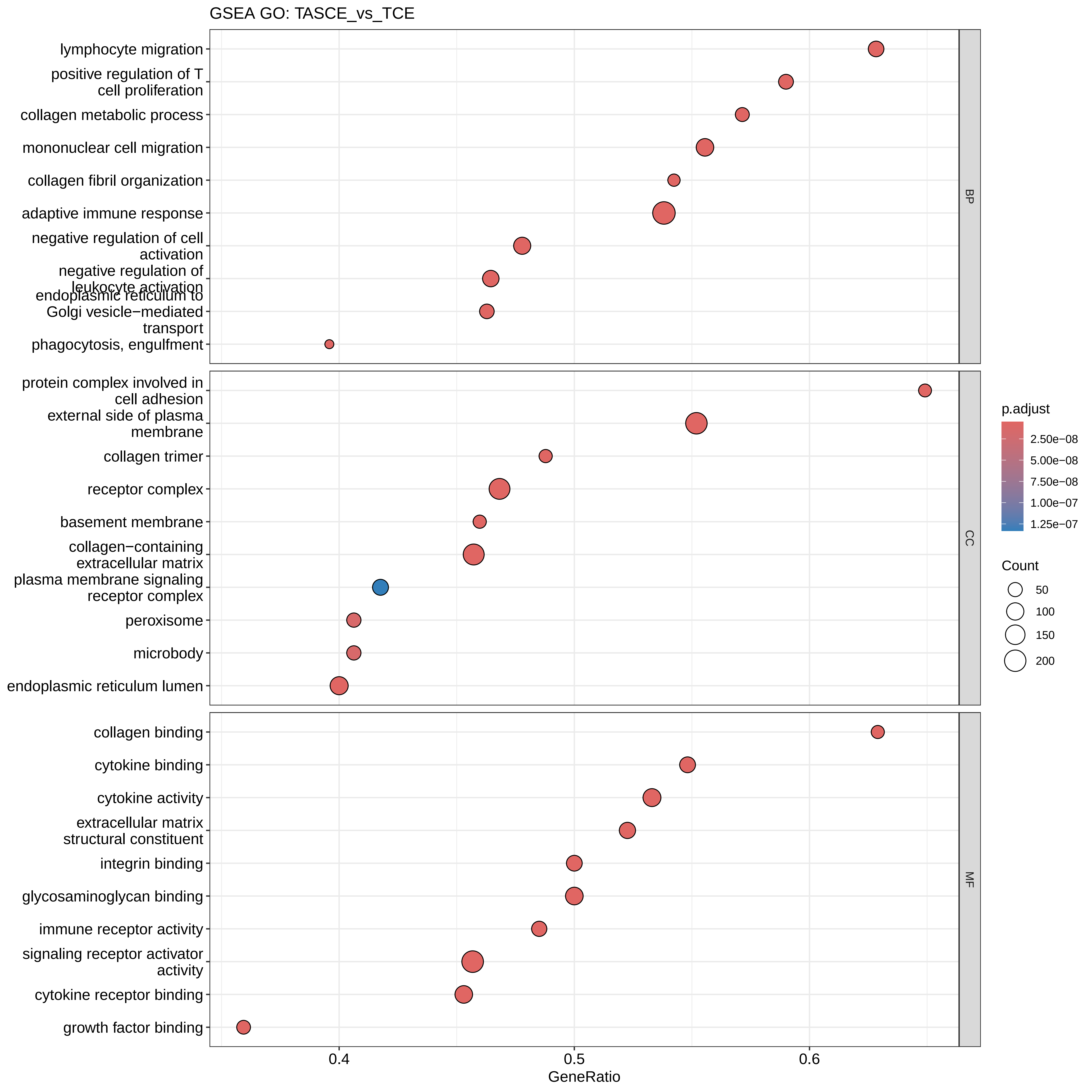
GSEA GO: TASCE_vs_TCE
lymphocyte migration
positive regulation of T cell proliferation
collagen metabolic process
mononuclear cell migration
collagen fibril organization
adaptive immune response
negative regulation of cell activation
negative regulation of leukocyte activation
endoplasmic reticulum to Golgi vesicle−mediated transport
phagocytosis, engulfment
BP
protein complex involved in cell adhesion
external side of plasma membrane
collagen trimer
receptor complex
basement membrane
collagen−containing extracellular matrix
plasma membrane signaling receptor complex
peroxisome
microbody
endoplasmic reticulum lumen
CC
collagen binding
cytokine binding
cytokine activity
extracellular matrix structural constituent
integrin binding
glycosaminoglycan binding
immune receptor activity
signaling receptor activator activity
cytokine receptor binding
growth factor binding
MF
0.4
0.5
0.6
GeneRatio
p.adjust
2.50e−08
5.00e−08
7.50e−08
1.00e−07
1.25e−07
Count
50
100
150
200

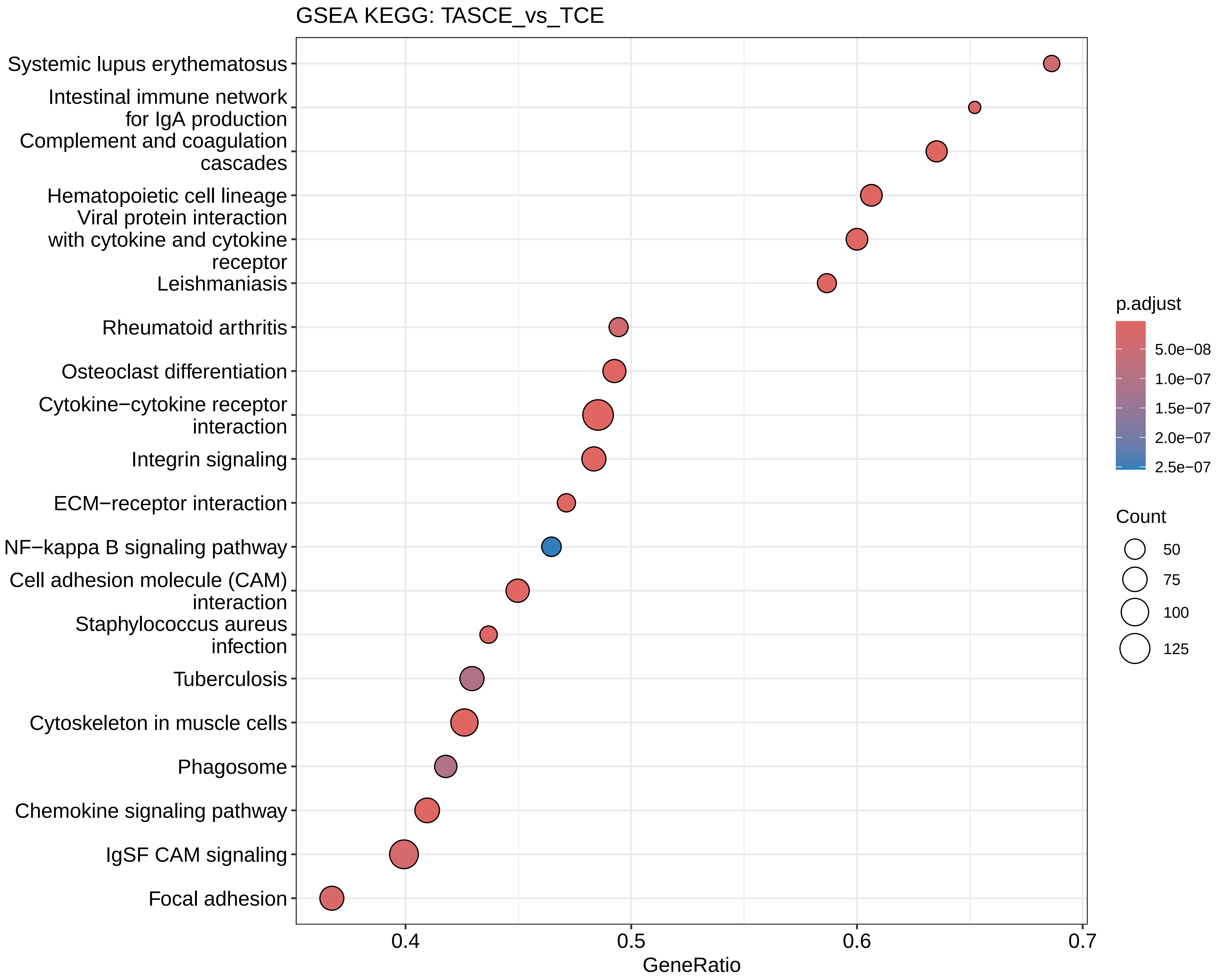
GSEA KEGG: TASCE_vs_TCE
Systemic lupus erythematosus
Intestinal immune network for IgA production
Complement and coagulation cascades
Hematopoietic cell lineage
Viral protein interaction with cytokine and cytokine receptor
Leishmaniasis
Rheumatoid arthritis
Osteoclast differentiation
Cytokine−cytokine receptor interaction
Integrin signaling
ECM−receptor interaction
NF−kappa B signaling pathway
Cell adhesion molecule (CAM) interaction
Staphylococcus aureus infection
Tuberculosis
Cytoskeleton in muscle cells
Phagosome
Chemokine signaling pathway
IgSF CAM signaling
Focal adhesion
0.4
0.5
0.6
0.7
GeneRatio
p.adjust
5.0e−08
1.0e−07
1.5e−07
2.0e−07
2.5e−07
Count
50
75
100
125

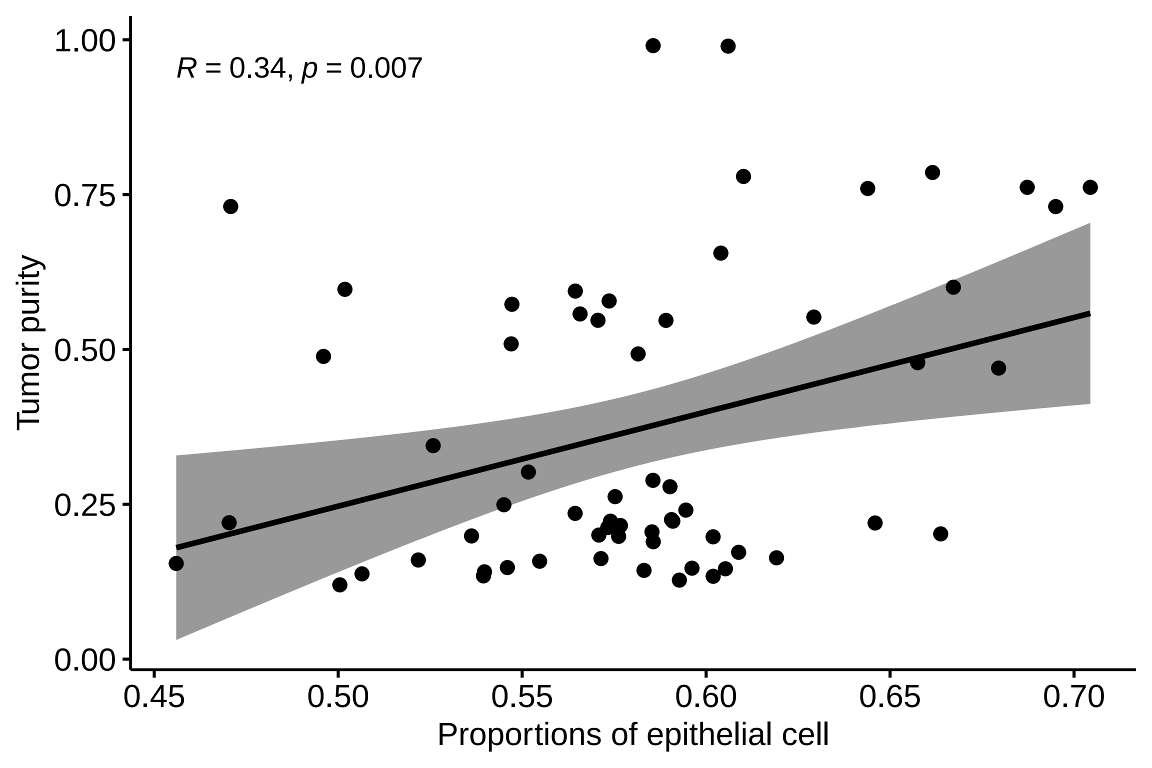

R = 0.34, p = 0.007
Tumor purity
1.00
0.75
0.50
0.25
0.00
0.45
0.50
0.55
0.60
0.65
0.70
Proportions of epithelial cell

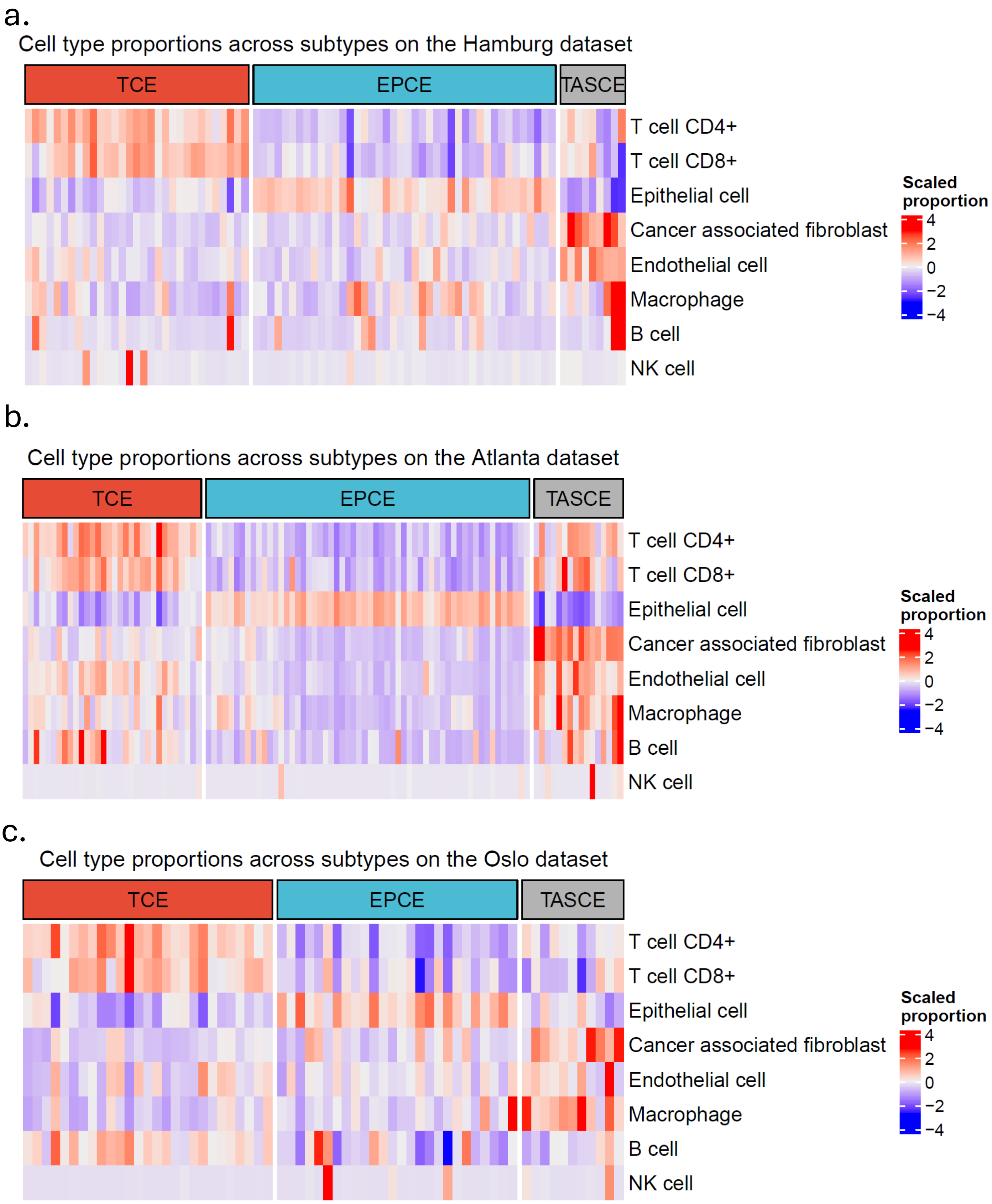

a.
Cell type proportions across subtypes on the Hamburg dataset
TCE
EPCE
TASCE
T cell CD4+
T cell CD8+
Epithelial cell
Cancer associated fibroblast
Endothelial cell
Macrophage
B cell
NK cell
Scaled proportion
4
2
0
−2
−4
b.
Cell type proportions across subtypes on the Atlanta dataset
TCE
EPCE
TASCE
T cell CD4+
T cell CD8+
Epithelial cell
Cancer associated fibroblast
Endothelial cell
Macrophage
B cell
NK cell
Scaled proportion
4
2
0
−2
−4
c.
Cell type proportions across subtypes on the Oslo dataset
TCE
EPCE
TASCE
T cell CD4+
T cell CD8+
Epithelial cell
Cancer associated fibroblast
Endothelial cell
Macrophage
B cell
NK cell
Scaled proportion
4
2
0
−2
−4

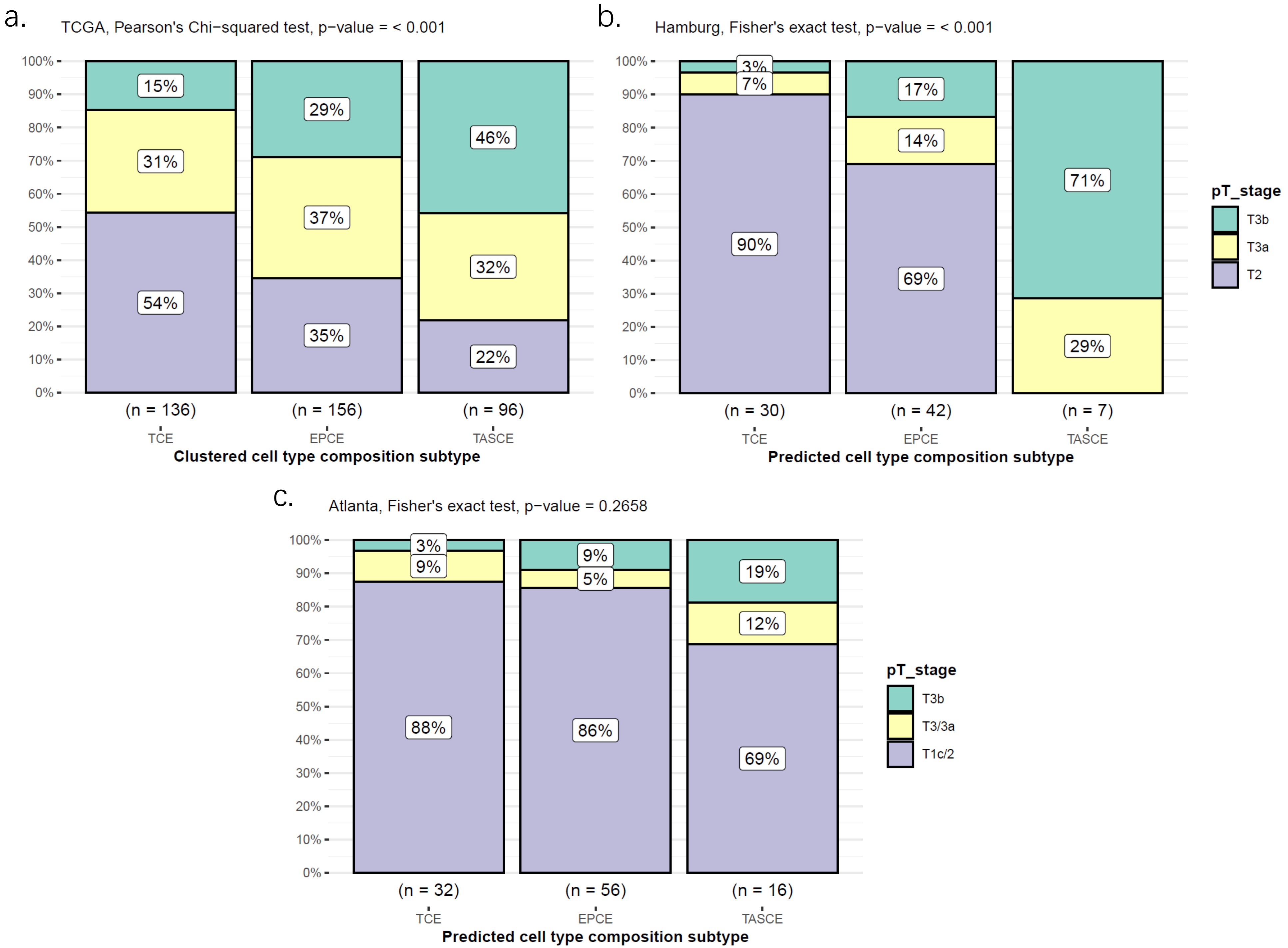
a.
TCGA, Pearson's Chi−squared test, p−value = < 0.001
15%
31%
54%
29%
37%
35%
46%
32%
22%
(n = 136)
(n = 156)
(n = 96)
TCE
EPCE
TASCE
Clustered cell type composition subtype
b.
Hamburg, Fisher's exact test, p−value = < 0.001
3%
7%
90%
17%
14%
69%
71%
29%
(n = 30)
(n = 42)
(n = 7)
TCE
EPCE
TASCE
Predicted cell type composition subtype
pT_stage
T3b
T3a
T2
c.
Atlanta, Fisher's exact test, p−value = 0.2658
3%
9%
88%
9%
5%
86%
19%
12%
69%
(n = 32)
(n = 56)
(n = 16)
TCE
EPCE
TASCE
Predicted cell type composition subtype
pT_stage
T3b
T3/3a
T1c/2

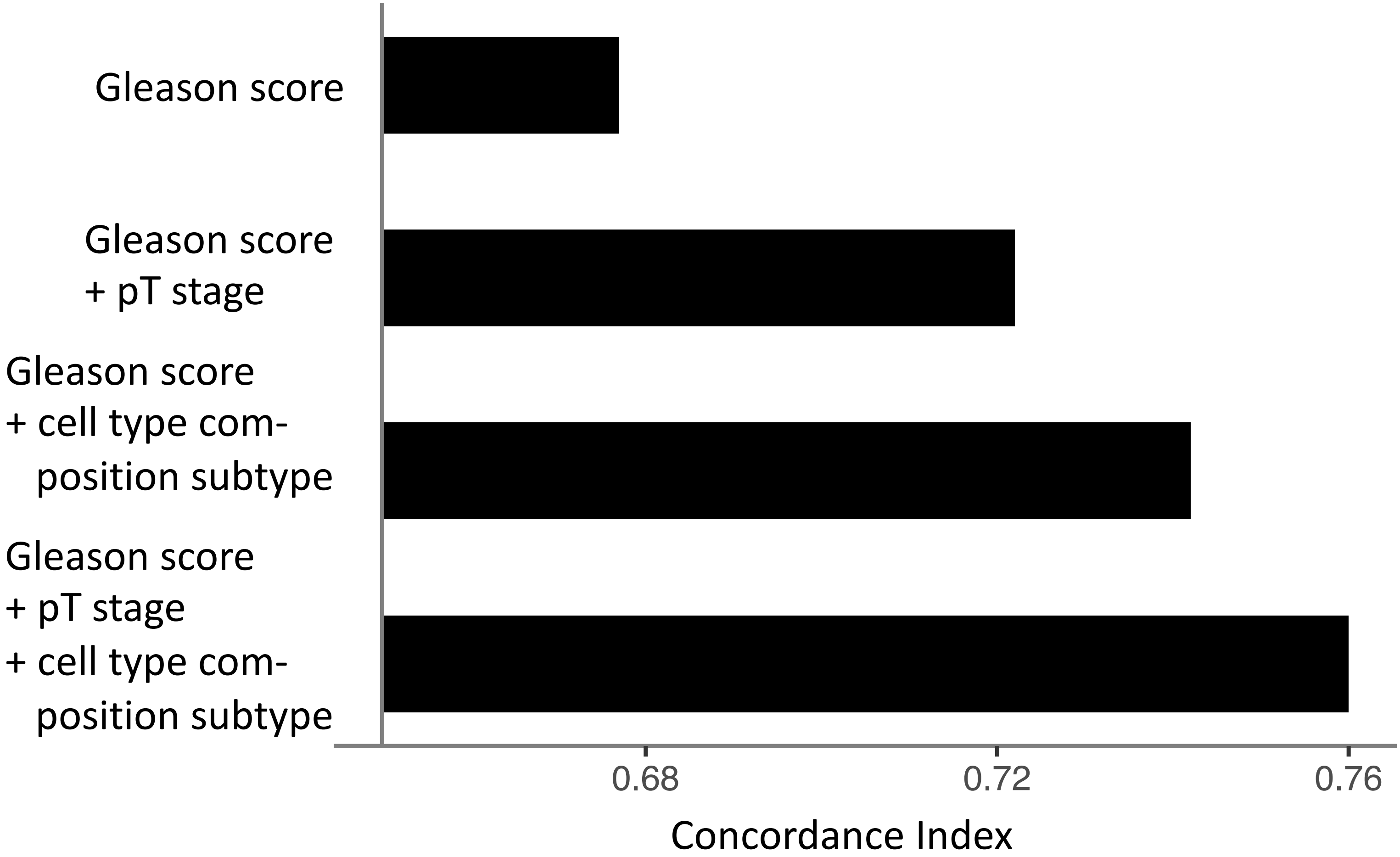
Gleason score
Gleason score + pT stage
Gleason score + cell type composition subtype
Gleason score + pT stage + cell type composition subtype
0.68
0.72
0.76
Concordance Index
